# Supplementary material for: Complement C3a signaling facilitates skeletal muscle regeneration by regulating monocyte function and trafficking
Source: Nat Commun. 2017 Dec 12;8:2078. doi: 10.1038/s41467-017-01526-z (PMC5727192; doi:10.1038/s41467-017-01526-z)
Supplement: Supplementary file 1 — Supplementary Information [file 41467_2017_1526_MOESM1_ESM.pdf]

Complement C3a signaling facilitates skeletal muscle regeneration by regulating monocyte function and trafficking

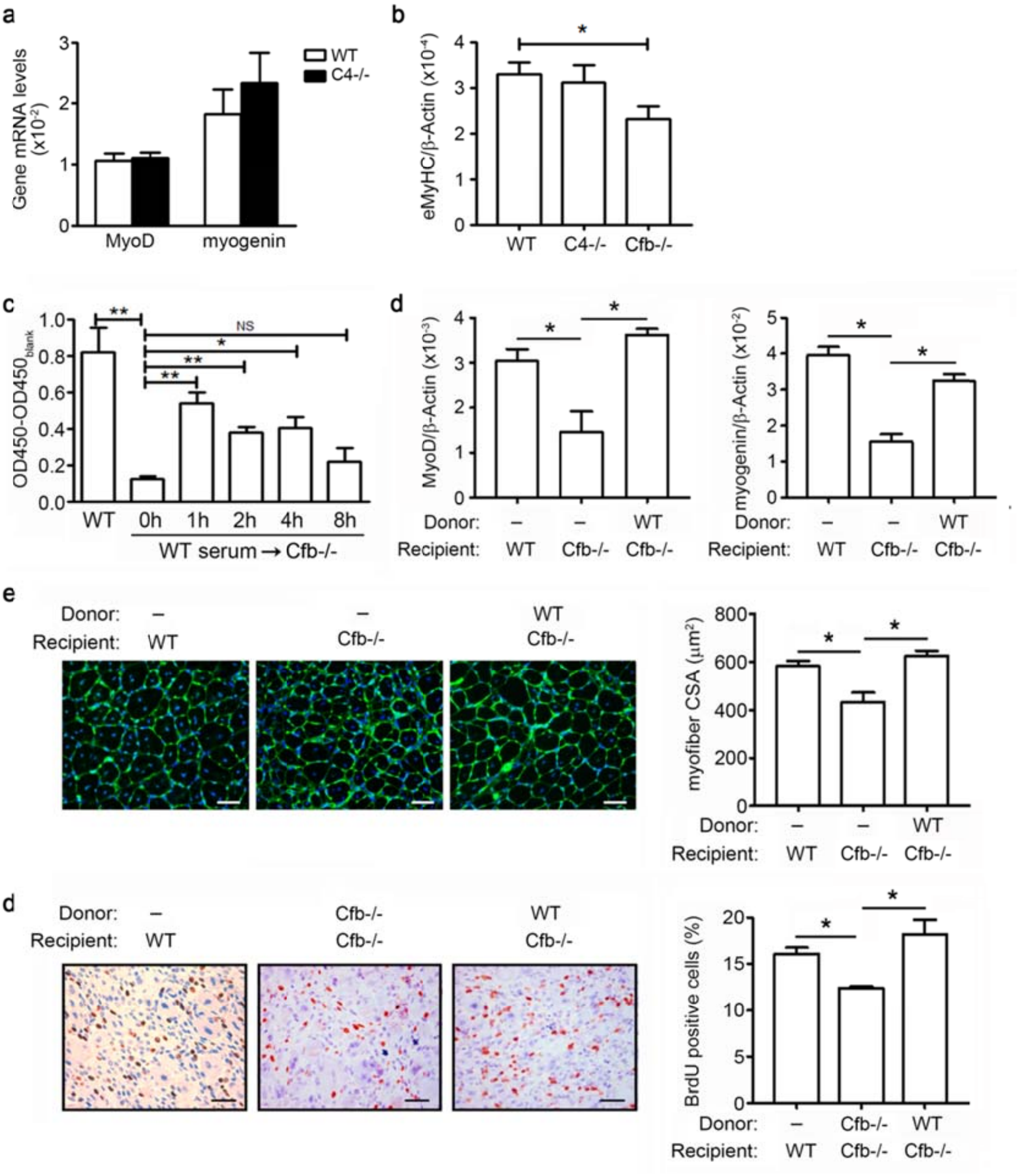

Supplementary Figure 1. Complement alternative activation is critical for muscle regeneration.

a. At day 5 after injury, the mRNA expression levels of MyoD and myogenin in WT and *C4-/-* muscle were assessed by realtime-PCR (N=4 in each group).

b. the mRNA expression levels of eMyHC in WT, *Cfb-/-* and *C4-/-* muscle were assessed by realtime-PCR (N=4 in each group).

c. WT mouse serum were injected to *Cfb-/-* mouse by intravenous, the recipient *Cfb-/-* mouse serum were collected at 1, 2, 4, 8 hours, then ELISA assay for LPS-dependent alternative pathway complement activity was performed.

d. At day 5 after CTX injury, the mRNA expression levels of MyoD and myogenin were assessed by realtime-PCR (N=4 in each group).

e. At day 15 after CTX injury, the mean myofiber cross section area (CSA) in injured muscles from WT mice, *Cfb-/-* mice and *Cfb-/-* mice with WT serum were measured. (N=4 in each group; bars, 50 μm)
f. BrdU immunohistochemical staining was used to detect the proliferating cells in injured muscle (at day 5 after injury) of WT mice, *Cfb-/-* mice and *Cfb-/-* mice with WT serum, the percentages of BrdU positive cell per field were analyzed. (N=4 in each group; bars, 50 μm).
Data are expressed as the mean ± s.e.m. *P<0.05 ,** P<0.01, unpaired t-test, two-tailed.

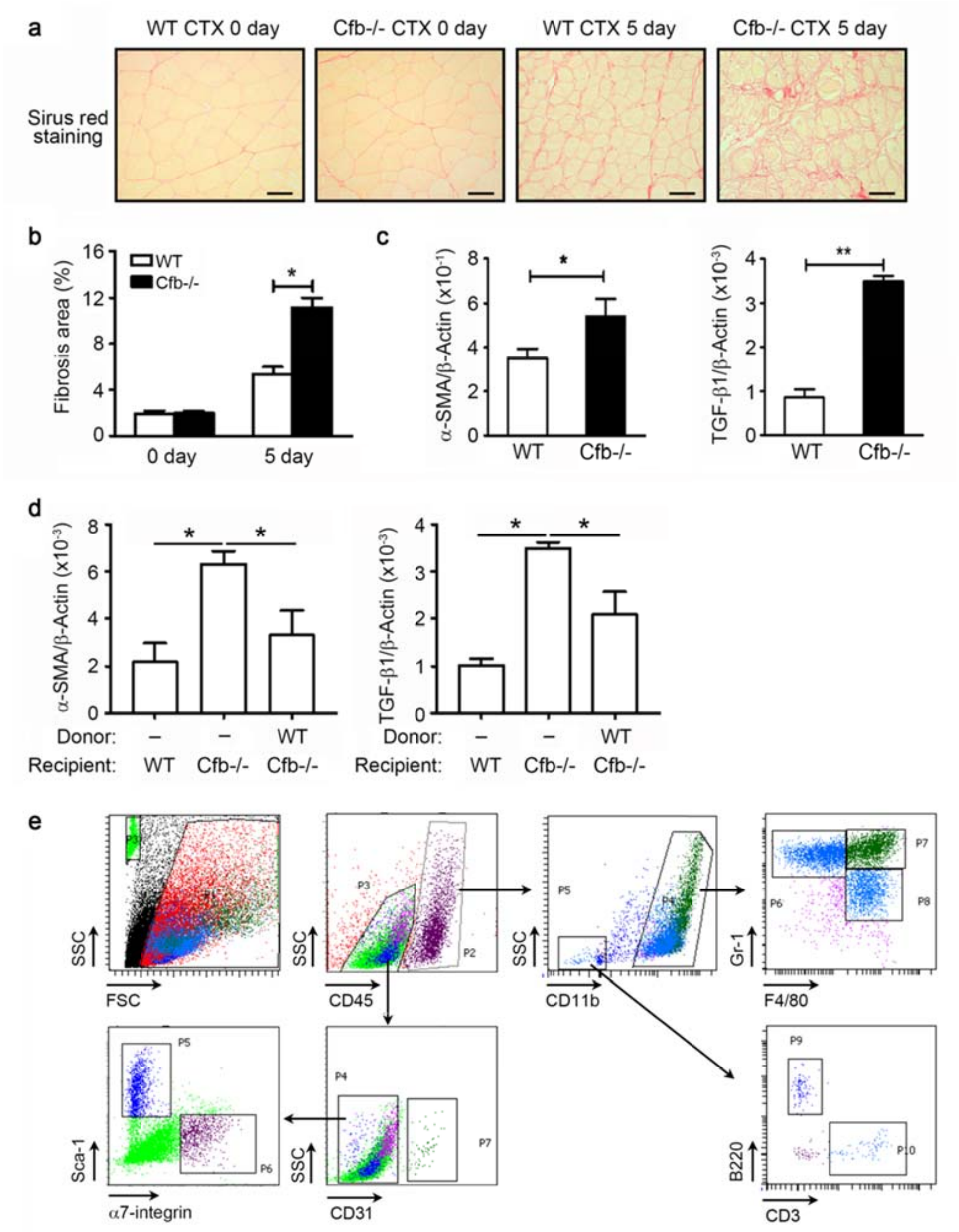

Supplementary Figure 2. Alternative pathway deficiency increases muscle fibrosis after injury,a. Sirus red staining was used to detect the collagen deposition (Red colour) in muscle (at day 0 and day 5 after injury) of WT and *Cfb-/-* mice. (N=4; bars, 50 µm)

b. The percentages of Sirus red staining positive area per field were analyzed. (N=4 in each group)

c. At day 5 after CTX injury, the mRNA expression levels of α-SMA and TGF-β1 in WT and *Cfb-/-* muscle were assessed by realtime-PCR (N=4 in each group).

d. At day 5 after CTX injury, the mRNA expression levels of α-SMA and TGF-β1 in WT, *Cfb-/-* and *Cfb-/-* with WT serum muscle were assessed by realtime-PCR (N=4 in each group).

e. FASC gating strategies. By anti-CD45 staining, cells were gated to CD45 positive bone marrow derived cells and CD45 negative muscle resident cells. Then by anti-CD11b, anti-CD3 and anti-B220 staining, cells in CD45 positive gate was divided into $CD11b^+$ monocytes cells, $CD3^+$ T cells and

$B220^+$ B cells. The cells in CD11b positive gate were further divided into $Gr1^+F4/80^-$ neutrophils, $Gr1^{hi}F4/80^+$ pro-inflammatory macrophages and $Gr1^{low}F4/80^+$ anti-inflammatory macrophages. By anti-CD31, anti-Sca-1 and anti-α7-integrin staining, cells in CD45 negative cells were divided into $CD31^+$ endothelia cells, $CD31^-Sca\text{-}1^+$ fibro/adipogenic progenitors and $CD31^-$α7-$integrin^+$ myoblast. Data are expressed as the mean ± s.e.m. $^*P<0.05$, $^{**}P<0.01$, unpaired t-test, two-tailed.

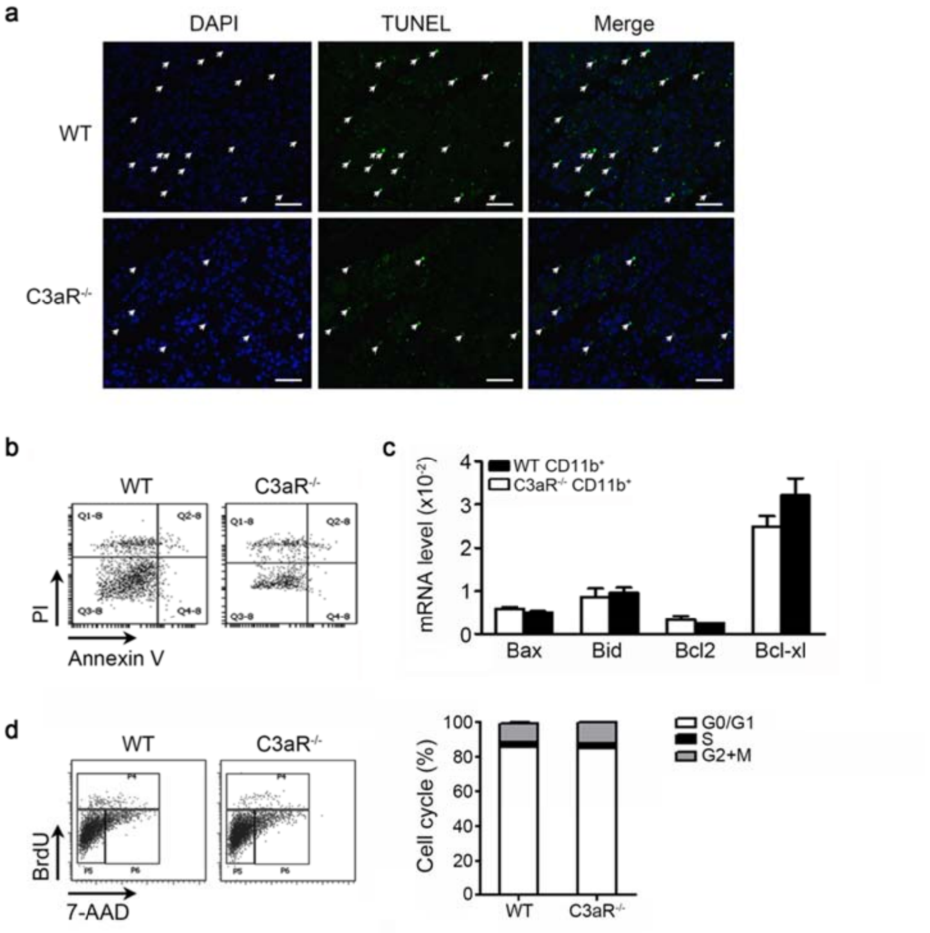

Supplementary Figure 3. C3aR deficiency dose not affect the proliferation and apoptosis of macrophages.

a. TUNEL staining was used to detect the apoptosis cells (green) in injured muscle (at day 3 after injury) of WT and *C3aR-/-* muscle, the nuclei were counter stained with DAPI (blue). (Bars, 50 μm).

b. At 3 days after CTX injury, the $CD45^{+}F4/80^{+}$ macrophages was gated, then Annexin V and PI analysis was used to detect the apoptosis ratio of WT and *C3aR-/-* macrophages.(N=3 in each group)

c. The mRNA expression levels of apoptosis associated gene Bax, Bid, Bcl2, Bcl-xl in sorted WT and *C3aR-/-* macrophages at 1 day after injury were accessed by realtime-PCR.(N=3 in each group)

d. The cell cycle of macrophages in WT and *C3aR-/-* muscle at day 2 after injury was accessed by BrdU staining. The macrophages was gated in $CD45^{+}CD11b^{+}F4/80^{+}$ cells.

Data are expressed as the mean ± s.e.m. *P<0.05, unpaired t-test, two-tailed.

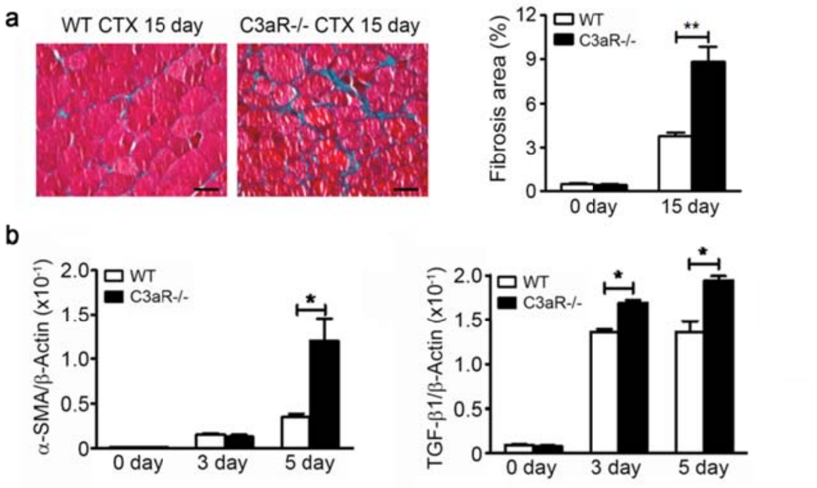

Supplementary Figure 4. Muscle fibrosis is increased in C3aR deficiency mouse.

a. Masson staining was used to detect the collagen deposition (green colour) in muscle (at day 15 after injury) of WT and *C3aR-/-* mice, the percentages of fibrosis area per field were analyzed. (Bars, 50 μm; N=4 in each group)

b. At 0, 3, 5 days after CTX injury, the mRNA expression levels of MyoD and myogenin in WT and *C3aR-/-* were assessed by realtime-PCR (N=4 in each group).

Data are expressed as the mean ± s.e.m. *P<0.05, unpaired t-test, two-tailed.

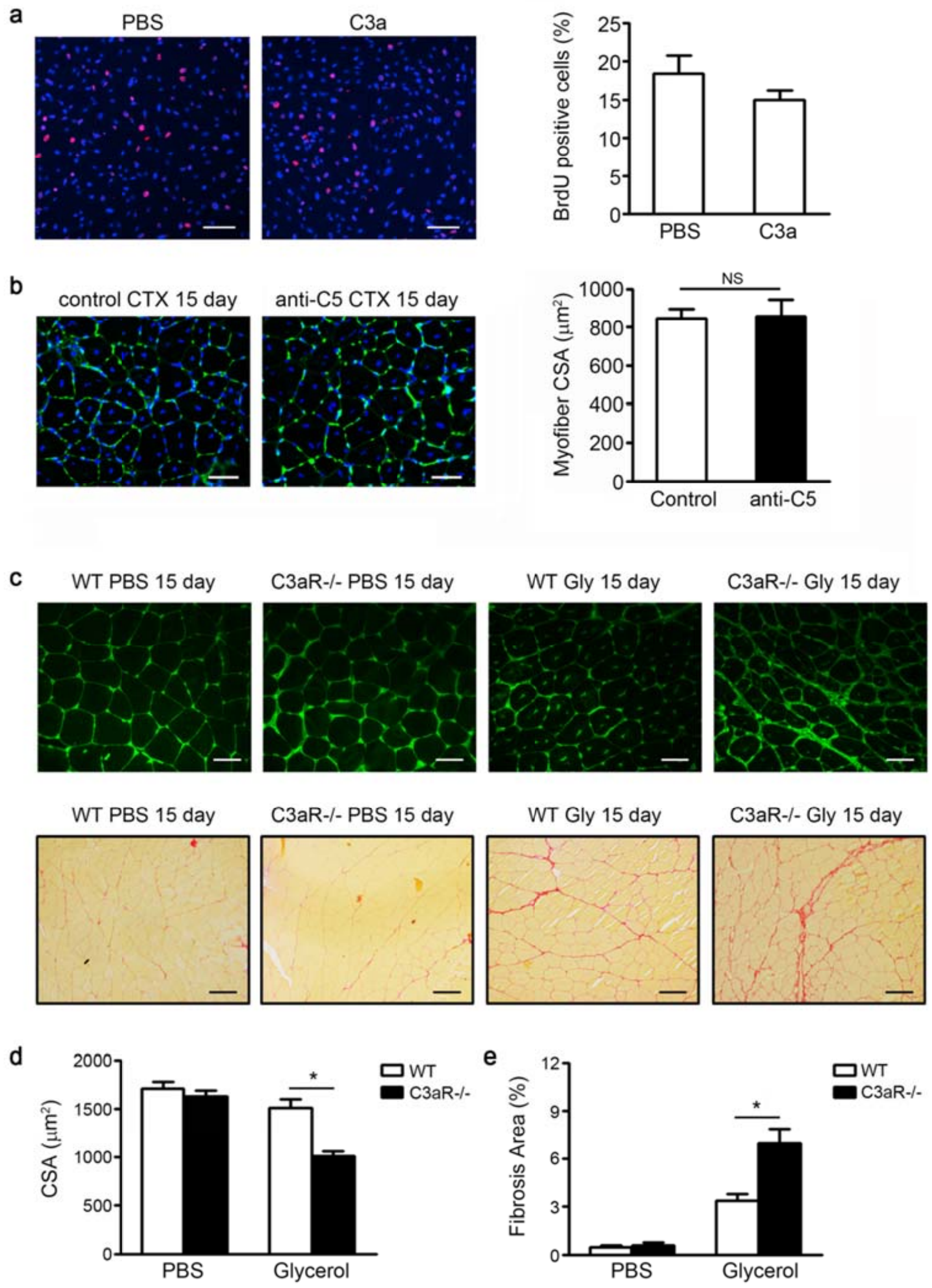

Supplementary Figure 5. Complement receptor C3aR deficiency impaires muscle regeneration.

a.The proliferation ratio of myoblast with or without C3a (10ng ml^{-1}) stimulation was detected by BrdU staining.(Bars,100 μm, N=6 in each group)

b. The anti-C5 monoclonal antibody and IgG control antibody was injected to WT mice (1mg/mice) by i.p.. At 15 day after CTX injury, muscle was immune-stained with WGA (green), the nuclei were counter stained with DAPI (blue) (Bars,50 μm); The mean myofiber cross section area (CSA) in injured muscles from IgG and anti-C5 muscle were measured. (N=4 in each group)

c. At 15 day after glycerol injury, the regeneration and fibrosis in WT and *C3aR-/-* muscle were examined by WGA staining (up panel; bars,50 μm) and Sirus Red staining (lower panel; bars,100 μm).

d. Graph was the statistics of myofiber mean CSA, ~250 myofiber per muscle (N=4 in PBS group, N=6

in Glycerol group)

e. Graph was the percentages of fibrosis area per field in WT and *C3aR-/-* muscle. (N=4 in PBS group, N=6 in glycerol group)

Data are expressed as the mean ± s.e.m. *P<0.05, unpaired t-test, two-tailed.

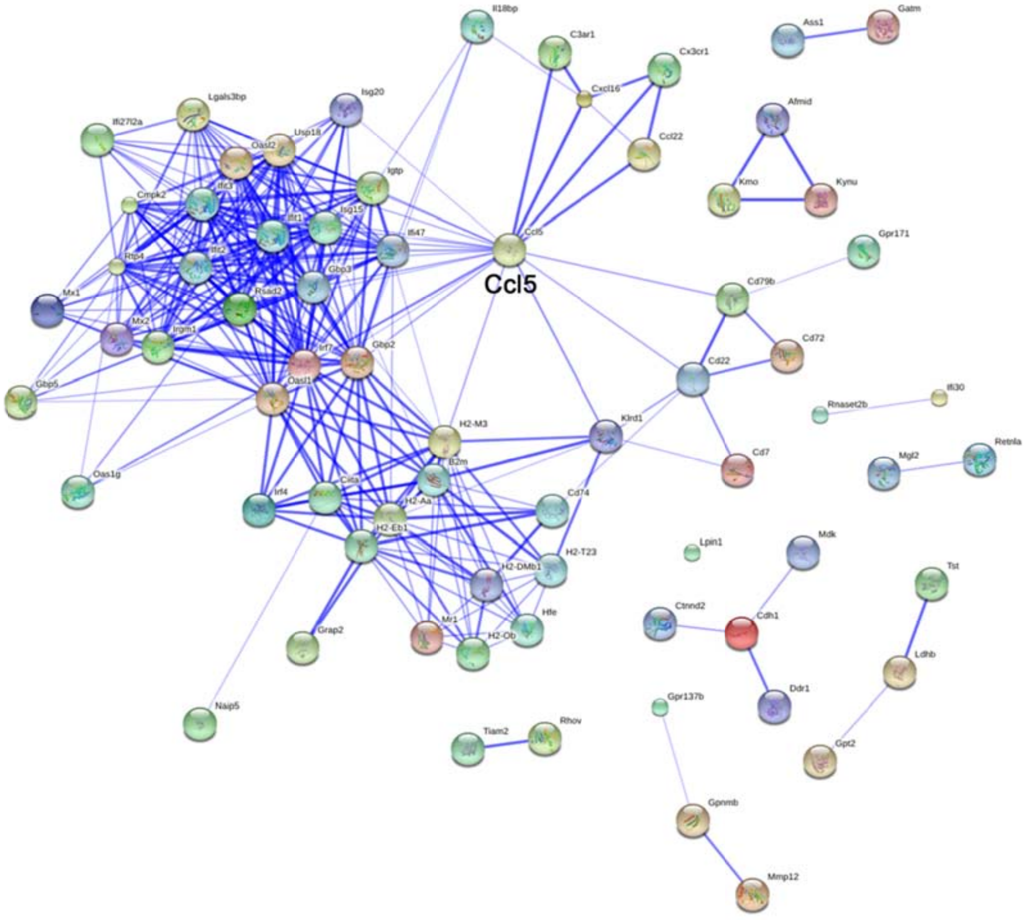

Supplementary Figure 6. The interaction network of down regulated genes in C3aR-/- macrophages is analyzed by STRING software.

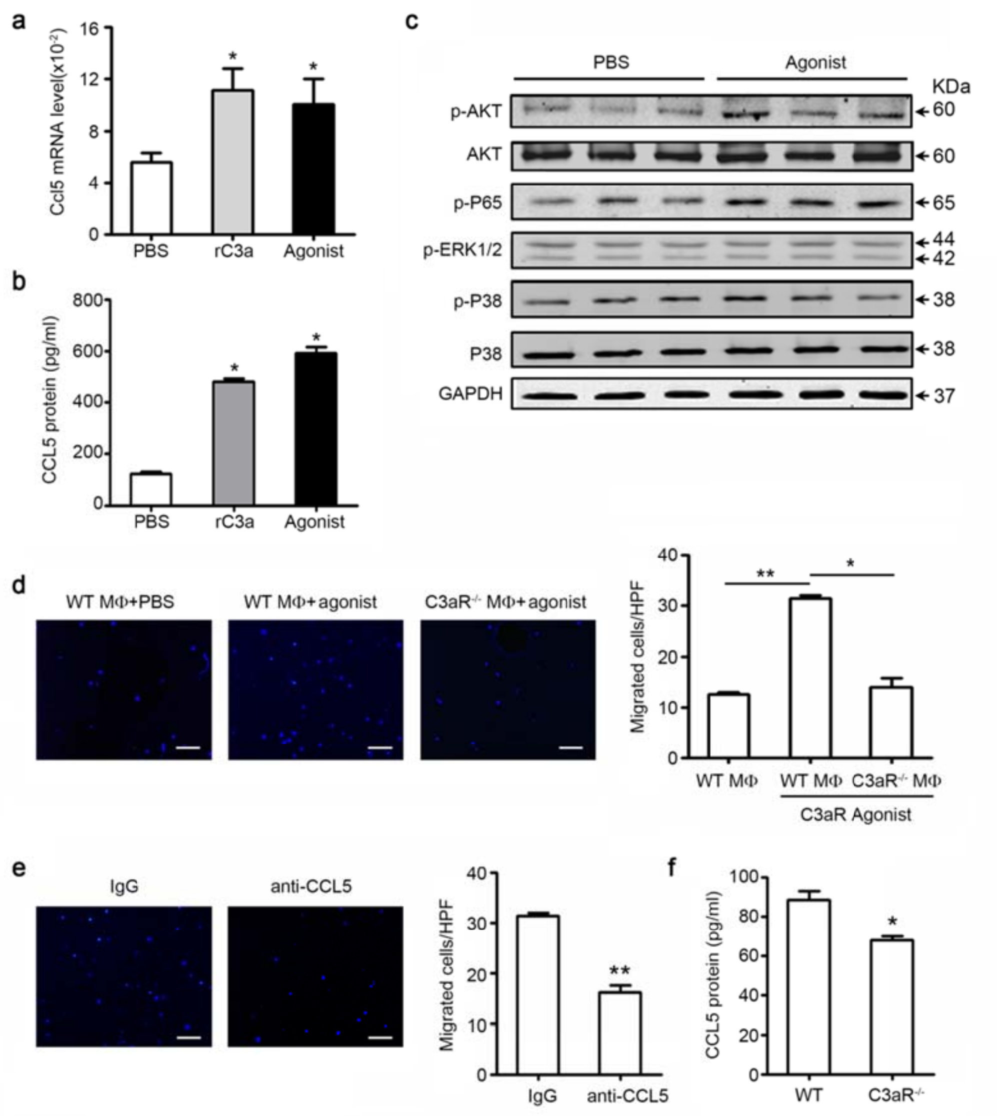

Supplementary Figure 7. C3a-C3aR pathway promotes the recruitment of macrophage by CCL5.

a. the mRNA levels of CCL5 in PBS, recombinant C3a (1ug/mL), C3aR agonist (1μM) stimulated macrophages were accessed by realtime-PCR. (N=4 in each group)

b. CCL5 ELISA assay was used to detect the concerntration of CCL5 in supernatant of PBS, recombinant C3a, C3aR agonist stimulated macrophages. (N=4 in each group)

c. The phosphorylation level of AKT, NF-κBp65 subunit, ERK1/2, MAPK p38 subunit in PBS or C3aR agonist stimulated macrophages (30min) was accessed by Western blot. (N=3 in each group)

d. WT or *C3aR-/-* bone marrow derived monocytes (5x10^5/well) with or without C3aR agonist (1μM) stimulation were seeded in the lower chamber and WT monocytes (5x10^4/well) were seeded in the upper chamber. 12 hours after co-culture, migrated cells from upper chamber were counted by DAPI staining (blue). (Bars, 50 μm, 10 filed was collected per sample, N=4 in each group,)

e. WT bone marrow derived monocytes (5x10^5/well) with C3aR agonist (1μM) stimulation were seeded in the lower chamber , CCL5 neutralization antibody(N=4) and IgG antibody (N=4) was added

in the supernatant, and WT monocytes ($5x10^4$/well) were seeded in the upper chamber. 12 hours after co-culture, migrated cells from upper chamber were counted by DAPI staining (blue). (Bars, 50 μm, 10 filed was collected per sample)

f. CCL5 concentration in the supernatant of WT (N=8)and *C3aR-/-* (N=4) muscle extract at day 1 after CTX injury was detected by CBA flex.

Data are expressed as the mean ± s.e.m. *P<0.05, ** P<0.01, unpaired t-test, two-tailed.

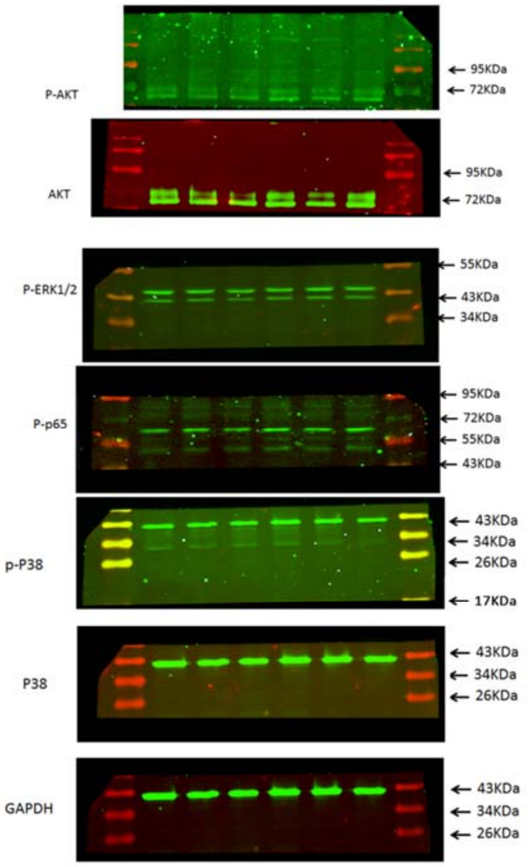

Supplementary Figure 8

Supplementary Figure 8. Uncropped Western blot scans of Supplementary Figure 7c.

Supplementary Table 1:

List of antibodies used in this study

Antibodies	Isotype	Dilution	Source
IHC and IF antibodies			
BrdU (Clone: BU-33)	Mouse monoclonal	1:2000	Sigma
C3b/iC3b (Clone:3/26)	Mouse monoclonal	1:50	Hycult Biotech
Western Blot antibodies			
Phospho-Akt (Ser473) (D9E)	Rabbit mAb	1:1000	Cell Signaling Technology
Akt (pan) (11E7)	Rabbit mAb	1:1000	Cell Signaling Technology
Phospho-NF-κB p65 (Ser468)	Rabbit mAb	1:1000	Cell Signaling Technology
Phospho-p38 MAPK (Thr180/Tyr182) (D3F9)	Rabbit mAb	1:1000	Cell Signaling Technology
p38 MAPK (D13E1)	Rabbit mAb	1:1000	Cell Signaling Technology
Phospho-p44/42 MAPK (Erk1/2) (Thr202/Tyr204) (D13.14.4E)	Rabbit mAb	1:1000	Cell Signaling Technology
GAPDH (D4C6R)	Mouse mAb	1:1000	Cell Signaling Technology
Flow cytometry antibody			
Antibody	Isotype	Dilution	Source
CD45 Percp Cy5.5(Clone:104)	Rat anti mouse	1:100	BD
CD11b APC Cy7(Clone:M1/70)	Rat anti mouse	1:100	BD
F4/80 PE(Clone:BM8)	Rat anti mouse	1:100	BD
Gr-1 FITC(Clone:R86-8C5)	Rat anti mouse	1:100	BD
CD3 PE-CF594(Clone:145-2C11)	Rat anti mouse	1:100	BD
B220 APC (Clone: RA3-6B2)	Rat anti mouse	1:100	BD
CD31 FITC(Clone:MEC13.3)	Rat anti mouse	1:100	BD
Sca-1 PE-Cy7(Clone:D7)	Rat anti mouse	1:100	BD
α7-integrin APC (Clone: 334908)	Rat anti mouse	1:10	R&D
FITC Annexin V Apoptosis Detection Kit II (Cat: 556570)			BD
APC BrdU Flow Kit (Cat: 552598)			BD
Secondary antibodies			
Antibody	Conjugate	Dilution	Source
Rabbit polyclonal anti-mouse IgG	Alexa 488	1:1000	Cell Signaling Technology

Supplementary Table 2:

List of primers used for qRT-PCR in this study

Gene	Accession Number	Forward primer	Reverse primer
C3aR	NM_009779	5'-CCCCAAGACATTGCCTCCAT-3'	5'-GACTGTGTTCACGGTCGTCT-3'
C5aR	NM_007577	5'-TCCTTCAGAAGAGTTGCCTGC-3'	5'-TTCTGTGGTAACCAGCGACG-3'
MyoD	NM_010866.2	5'-AGCATAGTGGAGCGCATCTC-3'	5'-GGTCTGGGTTCCCTGTTCTG-3'
Myogenin	NM_031189.2	5'-CAGCCCAGCGAGGGAATTTA-3'	5'-AGAAGCTCCTGAGTTTGCCC-3'
α-SMA	NM_007392	5'-TAACCCTTCAGCGTTCAGCC-3'	5'-ACATAGCTGGAGCAGCGTCT-3'
TGFb1	NM_011577.1	5'-GTCACTGGAGTTGTACGGCA-3'	5'-AGCCCTGTATTCCGTCTCCT-3'
eMHC	NM_001099635.1	5'-CTCTGTCACAGTCAGAGGTGT-3'	5'-TTCCGACTTGCGGAGGAAAG-3'
CD45	NM_011210	5'-CCTACACCCAGTGATGGTGC-3'	5'-TTGTGCTTGGAGGGTCAGTG-3'
F4/80	NM_008401	5'-CTGTGGACATGGACGCTGAT-3'	5'-GAGGCAAGGGACACACTGAC-3'
IL-1β	NM_008361	5'-CGTGGACCTTCCAGGATGAG-3'	5'-CATCTCGGAGCCTGTAGTGC-3'
IL-6	NM_031168.1	5'-GGTGACAACCACGGCCTTCCC	5'-AAGCCTCCGACTTGTGAAGTGGT-3'
CCL2	NM_011333	5'-GCACCAGCACCAGCCAACTCT-3'	5'-GCAGGCCCAGAAGCATGACAGG-3'
CCL3	NM_011337	5'-CTTGGAGGCAGCGAGGAACCC-3'	5'-AGCCCCTGCTCTACACGGGA-3'
CCL5	NM_013653.3	5'-TGCTCCAATCTTGCAGTCGT-3'	5'-TCTTCTCTGGGTTGGCACAC-3'
CXCL9	NM_008599.4	5'-CCAAGCCCCAATTGCAACAA-3'	5'-AGTCCGGATCTAGGCAGGTT-3'
CXCL16	NM_023158.6	5'-CAAGACCCTAGCGCCTACAG-3'	5'-TCACTGATGGAGACGAGCCT-3'
CD74	NM_010545.3	5'-GGCTCCACCTAAAGAGCCAC-3'	5'-GGGTGACTTGACCCAGTTCC-3'
H2-Ab1	NM_207105.3	5'-GTGTACCAGTTCATGGGCGA-3'	5'-ACGTACTCCTCCCGGTTGTA-3'
H2-Aa	NM_010378.2	5'-AGACACCCAGGGCCTTTATG-3'	5'-GAAGAGGGACACACGCCTTC-3'
H2-T23	NM_010398.3	5'-CCTGGACCGCGAATGACATA-3'	5'-GACTGCCTCTGACTTGTGCT-3'
CD22	NM_009845.3	5'-GCCCTTAAATTGATCCGTCAGG-3'	5'-CATGGTGTCTCGTCTGGGTG-3'
F11r	NM_172647.2	5'-AGCCAGATCACAGCTCCCTA-3'	5'-CATTGTCCTTCCGGGTCACA-3'
Cdh1	NM_009864.2	5'-AACCCAAGCACGTATCAGGG-3'	5'-GAGTGTTGGGGGCATCATCA-3'
Bax	NM_007527.3	5'-TGCTAGCAAACTGGTGCTCA-3'	5'-CACGGAGGAAGTCCAGTGTC-3'
Bid	NM_007544.3	5'-TCGCCCAAATAGGCGATGAG-3'	5'-GGGAAGGCTGTCTTCACCTC-3'
Bcl2	NM_009741.4	5'-AACATCGCCCTGTGGATGAC-3'	5'-AAACAGAGGTCGCATGCTGG-3'
Bcl-xL	NM_009743.4	5'-AGTAAACTGGGGTCGCATCG-3'	5'-GCCATCCAACTTGCAATCCG-3'
GAPDH	NM_008084	5'-CATGGCCTTCCGTGTTCCTA-3'	5'-GCGGCACGTCAGATCCA-3'
